# Antivascular Endothelial Growth Factor Agents for Neovascular Age-Related Macular Degeneration

**DOI:** 10.1155/2012/319728

**Published:** 2011-11-19

**Authors:** Ilias Zampros, Anna Praidou, Periklis Brazitikos, Panagiotis Ekonomidis, Sofia Androudi

**Affiliations:** Department of Ophthalmology, Aristotle University, 54124 Thessaloniki, Greece

## Abstract

Age-related macular degeneration (AMD) is the leading cause of severe visual loss and blindness over the age of 50 in developed countries. Vascular endothelial growth factor (VEGF) is considered as a critical molecule in the pathogenesis of choroidal neovascularization (CNV), which characterizes the neovascular AMD. Anti-VEGF agents are considered the most promising way of effectively inhibition of the neovascular AMD process. VEGF is a heparin-binding glycoprotein with potent angiogenic, mitogenic and vascular permeability-enhancing activities specific for endothelial cells. Two anti-VEGF agents have been approved by the US Food and Drug Administration (FDA) for the treatment of neovascular AMD. Pegaptanib sodium, which is an aptamer and ranibizumab, which is a monoclonal antibody fragment. Another humanized monoclonal antibody is currently off-label used, bevacizumab. This paper aims to discuss in details the effectiveness, the efficacy and safety of these three anti-VEGF agents. New anti-VEGF compounds which are recently investigated for their clinical usage (VEGF-trap, small interfering RNA) are also discussed for their promising outcomes.

## 1. Introduction

Vascular endothelial growth factor (VEGF) is a critical molecule involved in the pathogenesis of neovascular eye diseases such as neovascular age-related macular degeneration (AMD), proliferative diabetic retinopathy (PDR), diabetic macular edema (DME), retinal vein occlusion (RVO), and retinopathy of prematurity (ROP) [1].

Anti-VEGF-based therapies which are currently under investigation for their efficacy and safety in these neovascular diseases include an aptamer, pegaptanib sodium, ranibizumab and bevacizumab (VEGF-specific antibodies), the immunoglobulin G (IgG)-VEGF receptor fusion protein, VEGF trap, and the small interfering RNA (siRNA), bevasiranib [1].

Purpose of this paper is to summarize the current literature regarding the effectiveness of current anti-VEGF treatment for neovascular AMD.

## 2. Vascular Endothelial Growth Factor (VEGF)

The advent of antivascular endothelial growth factor (VEGF) treatments marks a major advancement in the treatment of angiogenic eye disease [1].

VEGF was isolated in the 1980s [2] initially as a tumour-derived factor that increased vascular permeability [3] and subsequently as an endothelial mitogen. It is a homodimeric glycoprotein and is a growth factor specific for endothelial cells [4]. It is a critical regulator of vasculogenesis and angiogenesis, as well as a potent inducer of vascular permeability [5]. It is a member of the platelet-derived growth factor (PDGF) family [6]. VEGF levels are increased by hypoxia, rendering VEGF-driven angiogenesis a hallmark response to low oxygen tension [7]. Its expression is upregulated by a number of growth factors, including epidermal growth factor, transforming growth factor-*α* and -*β*, keratinocyte growth factor, insulin-like growth factor-I, fibroblast growth factor, and platelet-derived growth factor, such that a variety of local interactions can modulate local VEGF concentrations [6, 8].

 Three receptor tyrosine kinases have been identified for VEGF: VEGF receptor (VEGFR) 1 (fms-like tyrosine kinase-1) has both positive and negative angiogenic effects; VEGFFR2 (fetal liver kinase-1 and kinase insert domain-containing receptor) is the primary mediator of the mitogenic, angiogenic, and vascular permeability effects of VEGF-A; VEGFFR3 mediates the angiogenic effects on lymphatic vessels [4, 9].

 The VEGF gene family consists of VEGF-A, VEGF-B, VEGF-C, VEGF-D, and placental growth factor (PlGF), which have different binding affinities for the three VEGF receptors [10, 11]. VEGF-A, which is the best studied, has been most strongly associated with angiogenesis and thus consists the target of most anti-VEGF treatments [12, 13]. VEGF-A signals through two receptor tyrosine kinases, VEGFR1 and VEGFR2, and is the only member of the VEGF gene family found to be induced by hypoxia [14]. VEGF-B selectively binds VEGFR1 and has a role in the regulation of extracellular matrix degradation and cell adhesion and migration. Both VEGF-C and VEGF-D bind VEGFR2 and VEGFR3 and regulate lymphangiogenesis, but VEGF-C can also be found involved in wound healing [15]. PlGF selectively binds VEGFR1 and is the most abundantly expressed VEGF family member in endothelial cells. PlGF may potentiate VEGF-A-induced endothelial cell proliferation, but on its own PlGF exerts only weak mitogenicity [14].

 Alternative exon splicing of the human VEGF-A gene results in at least four major biologically active isoforms, containing 121, 165, 189, and 208 aminoacids (five more are VEGF_145 _, VEGF_162 _, VEGF_165b_, VEGF_183_, and VEGF_206_) [16]. VEGF_165_ is the predominant isoform in the human eye and is a heparin-binding, homodimeric, 45-kDa glycoprotein that is secreted, although a substantial fraction is bound to the cell surface and to the extracellular matrix. Both VEGF_189_ and VEGF_208_ are strongly heparin-binding and are sequestered in the extracellular matrix. VEGF_121_ does not bind heparin and is secreted. VEGF_165_ appears to be the isoform responsible for pathological ocular neovascularization [17–19]. VEGF_121_ appears to be essential for normal retinal vascular function. All VEGF-A isoforms except VEGF_121_ contain a plasmin cleavage site and theoretically may be cleaved by plasmin to generate the smaller VEGF_110_ form [16, 20].

 The properties of VEGF can be summarized as follows: [2, 21, 22]

1. stimulator of angiogenesis;
2. potent inducer of vascular permeability and fenestration;
3. proinflammatory agent;
4. neuroprotective agent;
5. in neurodegenerative studies, displays neuroprotective effects under conditions of hypoxia, oxidative stress, and serum deprivation;
6. in an in vitro model of cerebral ischemia, reduces cell death;
7. in vitro, protects hippocampal, cortical, cerebellar granule, dopaminergic, autonomic, and sensory neurons;
8. vessel survival factor.

More extensively, we can comment that VEGF acts through various pathways which results in promoting pathologic neovascularization. It stimulates angiogenesis by being a potent endothelial cell mitogen and sustains endothelial survival by inhibiting apoptosis. In addition, VEGF is a chemoattractant for endothelial cell precursors, including their mobilization from the bone marrow and promoting their differentiation. Bone-marrow-derived endothelial cell precursors recruited in response to adenoviral-expressed VEGF_165_ are necessary and sufficient for tumor angiogenesis and are capable of inducing choroidal neovascularization (CNV) in mouse models. It is a powerful agonist of vascular permeability which is particularly important in CNV. Increased vascular permeability in response to VEGF may be due to formation of fenestrations in microvascular endothelium.

Leukocytes may amplify the effects of VEGF via their own secretion of VEGF. Furthermore, VEGF's proinflammatory activity, predominantly through the 164 isoform, contributes to pathological ocular neovascularization.

 VEGF has neuroprotective properties [23] that may be attributed in part to its ability to increase the survival of neurons and proliferating Schwann cells [24]. In rat models when administered intravitreally, it displayed a protective effect on apoptotic retinal cells in a dose-dependant manner. This protective effect was inhibited by blockade of all VEGF isoforms but not by blocade of VEGF_164_ alone.

 VEGF causes angiogenesis by indirect mechanisms too. Endothelial cell expression of metalloproteinases, which degrade the extracellular matrix and facilitate tissue invasion by new vessels, is upregulated by VEGF. Concurrently, endothelial cell expression of tissue inhibitors of metalloproteinases is downregulated in response to VEGF, which facilitates coordination of the angiogenic process. Also, VEGF increases the expression endothelial nitric oxide [25] synthase, which is an important mediator of VEGF-induced endothelial cell proliferation.

 The central role of VEGF-A is well established in ocular neovascular diseases [26–30]. High levels of VEGF-A expression are found in CNV tissue excised from patients with age-related macular degeneration (AMD). Intraocular VEGF-A levels correlate with blood vessel formation in patients with diabetic retinopathy and other retinal disorders [31–33].

 Different VEGF-A isoforms may have different functions in ocular diseases. VEGF_164_ is the predominant isoform expressed at the time of maximal preretinal neovascularization in a neonatal rat model and is the primary proinflammatory isoform in the retina of rats with diabetes. Levels of both VEGF_121_ and VEGF_165_ are increased in monkeys after laser-induced retinal vein occlusion. VEGF_120_ is the main isoform expressed in mouse CNV membranes, and inhibition of VEGF_120_ results in reduction of CNV in mice. Both VEGF_121_ and VEGF_165_ isoforms are found in CNV tissue excised from patients with AMD. Also, it seems that VEGF levels, in rat models, increased in direct proportion to the degree of neovascularization.

 In studies of autopsy eyes, VEGF levels were found to be elevated in the retinal pigment epithelium (RPE) and choroidal blood vessels of macula with AMD [26, 30, 34].

 The RPE is believed to play a key role in regulating VEGF levels. In ischemic retinal disorders, VEGF secretion by cultured RPE was found to be strikingly increased by hypoxia [2, 35].

Thus, we conclude that experimental elevation of VEGF levels leads to ocular neovascularization and that inhibition of VEGF action can prevent the development of ocular neovascularization. 

Preclinical studies have confirmed that increased expression of VEGF is both necessary and sufficient for inducing neovascularization, and several studies have provided evidence that specific inhibition of VEGF can inhibit neovascularization in the iris, choroid, cornea, and retina [36–38].

## 3. Anti-VEGF Agents

Inhibiting the activity of VEGF is believed to be an essential treatment strategy for many forms of ocular neovascularization [39]. Currently, mainly 3 anti-VEGF agents are in clinical use; ranibizumab (Lucentis), pegaptanib sodium (Macugen), and bevacizumab (Avastin) [1, 2].

 At this point, it must be noted that AMD is the leading cause of irreversible, severe loss of vision in people over the age of 50 in the developed world, and it remains an area of unmet medical need. The neovascular form of the disease represents approximately 10% of the overall disease prevalence, but it is responsible for 90% of the severe vision loss [40].

 Neovascular AMD is characterized by CNV that invades the subretinal space, often leading to exudation and hemorrhage. If the condition is remained untreated, damage to the photoreceptors and loss of central vision [41] usually results, and after several months to years, the vessels are largely replaced by a fibrovascular scar. Patients in whom a central scotoma develops have difficulty performing critical tasks that are typically associated with central vision, such as reading, driving, walking, and recognizing faces, and the difficulty has a major effect on their quality of life [42]. Thus, we can understand that the introduction of the anti-VEGF agents is a true revolution [43].

 Pegaptanib sodium (Macugen), first approved in the United States for the treatment of neovascular AMD in 2004, is a 28-base ribonucleic acid aptamer covalently linked to two branched 20-kD polyethylene glycol moieties, that was developed to bind and block the activity of extracellular VEGF, specifically at the 165-amino-acid isoform (VEGF_165_). 

 Ranibizumab (Lucentis) is a fragment of a recombinant, humanized, monoclonal antibody Fab that binds to and inhibits all the biologically active forms of vascular endothelial growth factor A (VEGFA) [44].

Bevacizumab (Avastin) is an anti-VEGF full-length antibody that initially was approved for the treatment of metastatic cancer of the colon or rectum. It is used as a possible off-label, safe alternative choice of lower cost [45]. It is the paternal molecule of ranibizumab.

 These agents are administered intravitreally pars plana [46–48].

## 4. Ranibizumab (Lucentis)

FDA has approved the use of ranibizumab as a treatment for all the angiographic subtypes of the subfoveal neovascularization of AMD. In clinical studies of phases I and II ranibizumab has shown encouraging results of biological activity with acceptable safety when it was administered intravitreously for six months to patients suffering from neovascuclar type of AMD.

 In phase III trial MARINA [44], patients suffering from neovascular type AMD were randomly assigned into 3 groups in a 1 : 1 : 1 ratio, receiving ranibizumab at a dose of either 0.3 mg or 0.5 mg or a sham injection monthly (within 23 to 37 days) for two years (24 injections) in one eye.

 The results were encouraging. After twelve months of treatment, the 94.5% of the patients receiving 0.3 mg and 94.6% of those receiving 0.5 mg every month had lost fewer than 15 letters ETDRS from baseline visual acuity. In the sham injection group the percentage dropped significantly to the level of 62.2%. At 24 months, this end point was met by 92.0% of the patients receiving 0.3 mg of ranibizumab and 90.0% of those receiving 0.5 mg, as compared with 52.9% in the sham injection group. The visual acuity benefit associated with ranibizumab was independent of the size of the baseline lesion, the lesion type, or baseline acuity.

 At 12 and 24 months, approximately 25% of patients treated with 0.3 mg of ranibizumab and 33% of patients treated with 0.5 mg of ranibizumab had gained 15 or more letters in visual acuity, as compared to 5% or less in the sham injection group.

 At both doses of ranibizumab, the mean improvement from baseline in visual acuity scores was evident 7 days after the first injection, whereas mean visual acuity in the sham injection group declined steadily over time at each monthly assessment. At 12 months, mean increases in visual acuity were 6.5 letters in the 0.3 mg group and 7.2 letters in the 0.5 mg group, as compared with a decrease of 10.4 letters in the sham injection group the benefit in visual acuity was maintained at 24 months.

 At baseline, the percentages of patients with 20/40 vision or better were similar among the three groups. At 12 months, approximately 40% of patients receiving ranibizumab had 20/40 vision or better, as compared with 11.3% in the sham injection group. At 24 months, of the patients receiving ranibizumab, 34.5% of those in the 0.3 mg group and 42.1% in the 0.5 mg group had at least 20/40 vision, whereas the proportion in the sham injection group had dropped to 5.9%.

Respectively, big differences were observed to the patients with baseline visual acuity 20/20 or better. After 12 months, the percentages were 3.8% for the 0.3 mg group, 7.9% for the 0.5 mg group, and 0.4% for the sham injection group.

 The percentages of patients with visual acuity of 20/200 or worse were similar among the 3 groups at baseline. At 12 and 24 months, the percentages in the ranibizumab-treated groups remained the same, whereas the percentages in the sham injection group had increased by 3 to 3.5 times.

 Very few patients receiving ranibizumab had severe loss of vision (30 letters or more) from baseline whereas in the sham injection group the percentages were 14.3% at 12 months and 22.7% at 24 months.

 Cumulated adverse events for the 24-month study period are summarized in Table 1 as well as the percentages of each adverse event [44].

 In conclusion, according to this phase III study, patients treated with ranibizumab had benefits. This study demonstrated not only prevention of vision loss but also a mean improvement, whereas patients in sham injection group continued to decline. These benefits are being achieved with low-rate appearance of severe ocular adverse events. The rate of the adverse events did not differ much from the rates in the sham injection group. The three treatment groups did not clearly differ in their rates of nonocular adverse events too.

Similar results and benefits for the patients suffering neovascular type of AMD presented the PIER study [49]. In this study, ranibizumab was administered at doses of 0.3 mg and 0.5 mg for three months followed by a fourth dose after a period of 3–12 months. On the same time, the rate of adverse events or side effects was low (both ocular and nonocular). Approximately, the 90% of the treated patients had lost fewer than 15 ETDRS letters compared with 50% of the sham group. In addition, at 12 months maintenance of visual acuity was accompanied on average by a significant reduction in vascular leakage, reduced foveal retinal thickness, and inhibition of CNV lesion growth. This initial impression is supported by individual OCT responses. Although this study provided clinically meaningful and statistically significant benefit to patients, the outcomes were not as strong as those observed with monthly dosing of ranibizumab, suggesting that some patients may require dosing on a more frequent schedule such as that used in the MARINA or ANCHOR studies. It is possible that OCT-guided administration of ranibizumab retreatment, as was used in a small, uncontrolled study, may allow greater individualization of ranibizumab dose intervals [50].

Also it would be interesting to compare ranibizumab with PDT treatment, a treatment modality frequently used so far ANCHOR a multicenter, double-masked, phase III trial compared patient-reported visual function in those with neovascular age-related macular degeneration treated with ranibizumab or verteporfin photodynamic therapy (PDT). Patients were randomized 1 : 1 : 1 to verteporfin PDT plus monthly sham intraocular injection or to sham verteporfin PDT plus monthly intravitreal ranibizumab (0.3 mg or 0.5 mg) injection. The need for PDT (active or sham) retreatment was evaluated every 3 months using fluorescein angiography (FA).

The conclusion of this study was that ranibizumab provided greater clinical benefit than verteporfin PDT in patients with age-related macular degeneration with new-onset, predominantly classic CNV. Rates of serious adverse events were low [51, 52].

The SUMMIT study goes one step beyond and tries to compare the combined therapy of PDT ranibizumab together versus ranibizumab alone. More specifically, SUMMIT is a clinical trial program that includes two similarly designed, controlled studies to further examine the safety, efficacy, and treatment burden of combination therapy with verteporfin photodynamic therapy (PDT) and ranibizumab compared with ranibizumab alone: DENALI in the USA, and Canada, examining verteporfin PDT in combination at both standard- and reduced-fluence light doses; MONT BLANC in Europe, examining verteporfin PDT in combination at standard-fluence light dose only. Twelve-month results of the MONT BLANC study show that combining standard-fluence PDT with ranibizumab 0.5 mg can deliver VA improvements that are noninferior to a ranibizumab monotherapy regimen with three ranibizumab-loading doses followed by injections on a monthly as-needed basis (noninferiority margin of 7 letters). There was no significant difference between the combination and monotherapy groups with regard to proportion of patients with a treatment-free interval of at least three months duration after Month 2. There were no unexpected safety findings, and adverse event incidence was similar between treatment groups.

Twelve-month results of the DENALI study showed that combining PDT with ranibizumab with three ranibizumab loading doses followed by additional injections on a monthly as-needed basis can improve visual acuity at month 12 in patients with subfoveal choroidal neovascularization (CNV) secondary to wet age-related macular degeneration (wet AMD). At month 12, patients in the standard fluence combination group gained on average 5.3 letters from baseline and patients in the reduced fluence combination group gained on average 4.4 letters. Patients in the ranibizumab monthly monotherapy group gained on average 8.1 letters at month 12. DENALI did not demonstrate non-inferior visual acuity gain for PDT combination therapy compared with ranibizumab monthly monotherapy.

Also some studies were conducted in order to check if the individualization of the treatment and the Pre Re Nata (PRN) (as needed) treatment especially under OCT guidance in patients with AMD can have the same efficacy and safety with the standard monthly injections. The SUSTAIN study [53], a twelve-month, phase III, multicenter, open-label, single-arm study concluded that the safety results are comparable to the favorable tolerability profile of ranibizumab observed in previous pivotal clinical studies; individualized treatment with less than monthly retreatments shows a similar safety profile as observed in previous randomized clinical trials with monthly ranibizumab treatment. Efficacy outcomes were achieved with a low average number of re-treatments. Visual acuity in SUSTAIN patients with individualized re-treatment based on VA/optical coherence tomography assessment reached on average a maximum after the first 3 monthly injections, decreased slightly under PRN during the next 2 to 3 months and was then sustained throughout the treatment period. Also PRONTO study [50], an open-label, single-center prospective study using a variable dosing regimen guided by optical coherence tomography (OCT) concluded that intravitreal injection of ranibizumab resulted in rapid improvements in visual acuity and OCT measurements. After 12 and 24 months, outcomes in the study were similar to the MARINA and ANCHOR phase III study results but with the mean frequency of dosing reduced by more than half, to about five injections per year. Based on these results, OCT appears to be a useful tool for guiding retreatment decisions for patients with neovascular AMD. Furthermore, the CATT study [54], a multicenter, single-blind, noninferiority trial, at year 1 showed that ranibizumab given as needed with monthly evaluation had effects on vision that were equivalent to those of ranibizumab administered monthly. Differences in rates of serious adverse events require further study.

## 5. Pegaptanib Sodium (Macugen)

Similarly, studies were conducted for pegaptanib sodium (Macugen) also. Different trials under the supervision of inhibition study in ocular neovascularization clinical trial group (VISION) were conducted [47].

 Patients with different types of CNV were separated into two groups. The first group was receiving a sham injection and the second one was receiving intravitreously an injection of pegaptanib sodium into one eye per patient every six weeks for a period of 48 weeks in total.

 In these trials, patients were receiving pegaptanib at a dose of 0.3 mg, 1.0 mg, 3.0 mg, or sham injection.

 In the combined analysis of the results of these trials and the three different doses of pegaptanib sodium, we can observe a significant difference between the patients receiving treatment and those receiving a sham injection.

 Pegaptanib sodium (Macugen) offered a statistically important and clinically essential benefit in the treatment of AMD. Declined risk for visual acuity loss observed to all doses only six weeks after the initiation of the treatment, the effectiveness of pegaptanib increased over time up to week 54, as measured by the mean loss of visual acuity from baseline to each study visit compared with that in the sham injection group. That is supported by many findings. Pegaptanib sodium reduced the risk not only of the visually acuity loss ≥15 ETDRS letters (considered as an average loss), but also of a loss ≥30 letters (considered as a severe loss). In addition, treatment with pegaptanib reduced the risk of progression to legal blindness, promoted stability of vision and in a small percentage of patients, resulted in more visual improvement at week 54 than among those receiving sham injections.

The visual results are further supported by angiographic measurements which suggested a reduction in the growth of the total size of the lesion or of CNV and in the severity of leakage. Also it causes reduction of the vascular permeability, factor that seems to have an important role in the improved visual outcomes observed. Because all forms of CNV have been associated with elevated levels of VEGF, it is hypothesized that a broad spectrum of patients might benefit with anti-VEGF therapy with pegaptanib sodium.

Most adverse events reported in the study eyes were transient, with a severity that was mild to moderate. Common ocular adverse events that occurred more frequently in the study eyes of patients treated with pegaptanib than in those receiving sham injection were eye pain (34% versus 28%), vitreous floaters (33% versus 8%), punctuate keratitis (32% versus 27%), cataracts (20% versus 18%), vitreous opacities (18% versus 10%), anterior chamber inflammation (14% versus 6%), visual disturbance (13% versus 11%), eye discharge (9% versus 8%), and corneal edema (10% versus 7%).

 The perinjection rates of adverse events were endophthalmitis (0.16%), retinal detachment (0.08%), and traumatic lens injury (0.07%), rates that were similar to the ones in the sham injection group. The risk of endophthalmitis was 1.3% per patient after one year of treatment.

Furthermore, there were no findings in relation to vital signs performed at each clinical assessment or ECG test results that were suggestive of a relationship to treatment with pegaptanib sodium; in particular, there was no evidence of an increase in mean blood pressure over the 3 years of treatment. The types and incidence of systemic serious adverse events observed are not unexpected in this elderly patient population, and none of these events was judged to be related to study drug. This favourable systemic safety profile is of particular importance for this population, that is, at higher risk for cardiovascular and thromboembolic diseases [55].

 In order to maximize the benefit of the treatment, it is critical that all treating ophthalmologists carefully adhere to an appropriate aseptic technique for each injection, educate patients regarding worrying symptoms, and closely monitor patients—after each injection.

 Similar results were achieved in vitro and in studies to animals.

Pegaptanib sodium was the first anti-VEGF agent approved for intravitreal administration for neovascular AMD. The VA results of the VISION study are clearly inferior to those of the MARINA and ANCHOR studies with ranibizumab. At the time of writing, therefore, pegaptanib sodium is considered second-line therapy. However, VA efficacy is only one of the clinical considerations that must be taken into account. Also the safety profile of the agent both in VISION study and in a german study [56] was good; VISION's safety profile was described earlier, and in the german study no relevant systemic or ocular side effects were noted. Cardiovascular incidents and overall mortality in the pegaptanib sodium group were comparable to those of the sham injection group. In addition, a separate trial looking at intravitreal pegaptanib sodium injections over a 2-year period concluded that there was no clinical- or angiographic-proven retinal or choroidal damage detectable. Thus, we understand that pegaptanib is a relatively safe choice that improves vision and must under consideration especially when the candidate of anti-VEGF treatment suffers from cardiovascular diseases.

 In conclusion, pegaptanib sodium offers, statically and clinically, a meaningful benefit to the patients suffering from AMD, regardless of the size or angiographic subtype of the lesion or the baseline visual acuity [1, 2, 40–50].

## 6. Bevacizumab (Avastin)

Bevacizumab is the father molecule of ranibizumab, and it is administered intravitreously as an off-label choice, since its original use is intravenous to colon and rectum cancers. Small study [39] by Ciulla and Rosenfeld has demonstrated benefits and results, for the patients suffering from AMD, similar to those of ranibizumab. Its rather smaller cost, consists a plus for using bevacizumab as treating agent [1, 2, 40–50].

## 7. Bevacizumab-Ranibizumab Relation

Due to the close molecular relation between bevacizumab (Avastin) and ranibizumab, a comparison between these two anti-VEGF agents would have been very interesting [54]. Also the lack of a large-scale clinical trial data (the small study by Ciulla and Rosenfeld) and the fact that in the United States bevacizumab is the most commonly used anti-VEGF agent by the ophthalmologists as an off-label, low cost alternative treatment, suggests the comparison between them even more necessary. This gap was recently filled by ranibizumab and bevacizumab for age-related macular degeneration study of the CATT research group.

 The purpose of this study was to assess the relative efficacy and safety of ranibizumab and bevacizumab and to determine whether an as-needed regimen would compromise long-term visual acuity, as compared with a monthly regimen.

 Patients were randomly assigned to four groups after the first mandatory intravitreal injection: ranibizumab every 28 days (ranibizumab monthly), bevacizumab every 28 days (bevacizumab monthly), ranibizumab only when signs of active neovascularization were present (ranibizumab as needed), and bevacizumab only when signs of active neovascularization were present (bevacizumab as needed).

 The dose was 0.5 mg (in 0.05 mi of solution) for ranibizumab and 1.25 mg (in 0.05 mL of solution) for bevacizumab. The commercially acquired bevacizumab had to be repackaged in glass vials in an aseptic filling facility.

 Every 28 days, patients in the groups that received study drugs as needed underwent time-domain OCT and were evaluated for treatment. Also fluorescein angiography was performed at the discretion of the ophthalmologists in order to aid in retreatment decisions.

 According to this study, visual acuity improved from baseline to 1 year in all four study groups. Most of the improvement occurred during the first six months. At 1 year, bevacizumab was equivalent to ranibizumab, both when the drugs were administered monthly and when the drugs were given as needed. Ranibizumab given as needed was equivalent to bevacizumab given monthly. The comparison between bevacizumab given as needed and ranibizumab given monthly was inconclusive, and so was the comparison between bevacizumab given as needed and bevacizumab given monthly.

 The outcomes for the pairwise comparisons between study groups did not change after the clinical adjustment for clinical center, age, baseline lesion size.

 At 1 year, the proportion of patients who did not have a decrease in visual acuity of 15 letters or more from baseline was 94.4% in the ranibizumab-monthly group, 94.0% in the bevacizumab-monthly group, 95.4% in the ranibizumab-as-needed group, and 91.5% in the bevacizumab-as-needed group. The proportion of patients who gained at least 15 letters increased during the first 36 weeks in all four study groups at 1 year did not differ significantly, ranging from 24.9 to 34.2%.

 The two drugs resulted in a substantial reduction in total retinal thickness at the fovea after the first injection. At four weeks, no fluid was detected on OCT for 27.5% of the patients treated with ranibizumab and 17.3% for patients treated with bevacizumab. At 1 year the proportion of patients with no fluid on OCT ranged from 19.2% among patients who received bevacizumab as needed to 43.7% among those who received ranibizumab monthly.

Dye leakage was absent on angiography in 58.8% of patients in the ranibizumab-monthly group, 57.7% in the bevacizumab-monthly group, 46.7% in the ranibizumab-as-needed group, and 41% in the bevacizumab-as-needed group. 

 However, the lesions size on angiography were slightly larger in the two groups treated as needed.

 The ocular adverse events were endophthalmitis (0.7% for ranibizumab monthly group and 1.4 for bevacizumab monthly group) and uveitis, retinal detachment, ocular-vessel occlusion or embolism, retinal tear, vitreous hemorrhage that each of them occurred in less than 1% of the patients.

 The serious systemic adverse events included arteriothrombotic events with a similar percentage of 2-3% for each group, venous thrombotic events in approximately 1.0% of the patients. One or more serious systemic adverse events occurred in 21.5% of patients in total, the percentages, respectively, for each group were 17.6% in the ranibizumab-monthly group, 22.4% in the bevacizumab-monthly group, 20.5% in the ranibizumab-as-needed group, and 25.7% in the bevacizumab-as-needed group.

 Other serious systemic adverse events with similar rates for group were cardiac disorder (3.3% to 5.6%), infection (2.0% to 6.0%), nervous system disorder (2.0% to 4.0%), injury or procedural complication (2.3% to 3.8%), benign or malignant neoplasm (1.7% to 3.4%), surgical or medical procedure (1.3% to 2.7%), and gastrointestinal disorder (0.7% to 3.0%).

 The comparison between the two drugs shown that these two anti-VEGF agents had equivalent effects on visual acuity at time points throughout the first year of followup. The mean number of letters gained the proportion of patients in whom visual acuity was maintained (<15 letters lost), and the proportion of patients who had a gain of at least 15 letters were nearly the same for each drug, when regimen was the same.

 Also excellent results for visual acuity could be achieved with less than monthly regimens for both drugs. The mean number of letters gained were 5.9 letters with bevacizumab given as needed, 6.8 letters with ranibizumab given as needed, 8.5 letters with ranibizumab-monthly, and 8.0 letters with bevacizumab-monthly.

 Both bevacizumab and ranibizumab substantially and immediately reduced the amount of fluid in or under the retina. The proportion of patients who had complete resolution of fluid was greater with ranibizumab than with bevacizumab. This difference was evident after first injection, with no fluid seen at 4 weeks in 27.5% of patients receiving ranibizumab and 17.3% of those receiving bevacizumab, and the difference persisted throughout the year. Furthermore, the absolute between drug differences in the amount of residual fluid was small, and in the majority of patients neither drug eliminated all fluid.

 The mean decrease in central retinal thickness was greater in the ranibizumab-monthly group (196 *μ*m) than in the other groups (152 to 168 *μ*m). 

 Monthly injections of either drug resulted in no increase in the mean lesion area, whereas there was a small increase when injections were given as needed.

 The rates of both ocular and nonocular adverse events were similar. However, the rate of serious systemic adverse events and primarily hospitalizations was higher among bevacizumab-treated patients than among ranibizumab-treated patients (24.1% versus 19.1%). But at this point it should be noted that the median age of patients in the CATT Study was over 80 years, and a high rate of hospitalizations might be anticipated as a result of chronic or acute medical conditions more common to older populations. 

After the head to head comparison between the two agents according to this study, we observe similar therapeutic results and adverse events. The factor that was significantly different was the average cost of drug per patient. It was 23,400 USD in ranibizumab-monthly group and 13,800 USD in ranibizumab as-needed group while in the bevacizumab groups it was 595 USD and 385 USD in bevacizumab-monthly and bevacizumab as needed, respectively. Previously it was mentioned that AMD is the leading cause of irreversible, severe loss of vision of people about 55 years of age or older thus resulting in a big number of patients. So this significant difference of treatment cost must go under serious consideration [57].

## 8. VEGF-TRAP

VEGF-Trap is a novel anti-VEGF drug. It is a receptor decoy that functions by literally trapping VEGF. It is a recombinant chimeric molecule that contains the VEGF-binding elements from the extracellular domains of VEGF receptors 1 and 2 fused to the Fc portion of human IgG. It binds all VEGFA isoforms and placental growth factor with high affinity [58].

 In several mouse models, VEGF-Trap was shown to partially suppress CNV when injected either subcutaneously (systemically) or intravitreally. It decreased the area of CNV in a mouse model of laser-induced CNV. It also decreased the area of CNV in transgenic mice that express VEGF in the photoreceptors. Subcutaneous injection of VEGF-TRAP led to a reduction in retinal vascular permeability in mice whose retinas were exposed to VEGF.

 Both aflibercept (the oncology product) and VEGF Trap-Eye are manufactured in bioreactors from industry standard Chinese hamster ovary cells that overexpress the fusion protein. However, VEGF Trap-Eye undergoes further purification steps during manufacturing to minimize risk of irritation to the eye. The highest intravitreal dose being used in pivotal trials for VEGF Trap-Eye is 2 mg/month [58].

 The safety, tolerability, and biological activity of intravitreal VEGF Trap-Eye in treatment of neovascular AMD was evaluated in the two-part Clinical Evaluation of Antiangiogenesis in the Retina-1 (CLEAR-IT-1) [59] study. The first part was a sequential cohort dose-escalation study in which 21 patients were monitored for safety, changes in foveal thickness on OCT, best corrected visual acuity (BCVA) and lesion size on FA for 6 weeks. No adverse systemic or ocular events were noted and visual acuity remained stable or improved ≥3 lines in 95% of patients with a mean increase in BCVA of 4.6 letters at 6 weeks. Patients showed substantially decreased foveal thickness.

 In the second part, patients received a single intravitreal injection of either 0.5 or 4 mg of VEGF Trap-Eye and were followed for 8 weeks. At 8 weeks, the mean decrease in retinal thickness in the low-dose group was 63.7 *μ*m compared to 175 *μ*m for the high-dose group.

 CLEAR-IT-2 trial was a prospective, randomized, multi-center, and controlled dose- and interval-ranging Phase II trial in which 157 patients were randomized to five-dose groups and treated with VEGF Trap-Eye in one eye. Two groups received monthly doses of either 0.5 or 2.0 mg for 12 weeks (at weeks 0, 4, 8, and 12) and three groups received quarterly doses of either 0.5, 2.0, or 4.0 mg for 12 weeks (at weeks 0 and 12). Following this fixed dosing period, patients were treated with the same dose of VEGF Trap-Eye. Patients initially treated with 2.0 or 0.5 mg of VEGF Trap-Eye monthly achieved mean improvements of 9.0 and 5.4 ETDRS letters with 29 and 19% gaining, respectively, ≥15 ETDRS letters at 52 weeks. Patients in these two monthly-dosing groups also displayed mean decreases in retinal thickness versus baseline of 143 *μ*m in the 2.0 mg group and 125 *μ*m in the 0.5 mg group at 52 weeks as measured by OCT. Patients in the three quarterly dosing groups also showed mean improvements in BCVA and retinal thickness; however, they were generally not as profound as the monthly injection group.

In phase III studies' VIEW 1 (conducted in USA and Canada) and VIEW 2 (Europe, Asia pacific, Latin America, and Japan) VEGF Trap-Eye was evaluated for its effect on maintaining and improving vision when dosed as an intravitreal injection on a schedule of 0.5 mg monthly, 2 mg monthly, or 2 mg every two months (following three monthly loading doses), as compared with intravitreal ranibizumab administered 0.5 mg every month during the first year of the studies. As-needed dosing with both agents, with a dose administered at least every three months (but not more often than monthly), is being evaluated during the second year of each study.

 The results for the primary and the first secondary endpoint prespecified for testing concerning both the maintenance (loss of 15 letters or less) and the mean improvement in vision at week 52 versus baseline are promising and similar at all groups. The percentages for maintenance are ranging from 94.4% to 96.3%, and the mean improvement is ranging from 6.9 to 10.9 letters. 

 Based on Phase II study data, VEGF Trap-Eye seems to be generally well tolerated with no serious drug-related adverse events. The most common adverse events reported in the study included conjunctival hemorrhage (38.2%), transient increased intraocular pressure (18.5%), refraction disorder (15.9%), retinal hemorrhage (14.6%), subjective visual acuity loss (13.4%), vitreous detachment (11.5%), and eye pain (9.6%) [60].

## 9. RNA Interference

SIRNA stands for short interfering RNA. SIRNAs are 21 to 25 nucleotide-long double-stranded RNA molecules capable of destroying a corresponding target messenger RNA with high selectivity and efficacy [61]. This leads to posttranscriptional gene silencing (PTGS).

 SIRNAs work intracellular where they are incorporated into a protein complex called RNA-induced silencing complex (RISC) [61]. The RISC has RNA helicase activity, which unwinds the two strands of RNA starting by the terminus with the lowest melting temperature (highest A-U base pairs). 

 The strand of the siRNA that becomes associated to the RISC leads the complex to selectively cleave and degrade messenger RNA molecules containing a complementary sequence. The siRNA is engineered to match the protein encoding nucleotide sequence of the target messenger RNA. Since the translation of messenger RNA into proteins is an amplification step whereby a single messenger RNA molecule can lead to the production of around 5.000 copies of a protein molecule, destroying the messenger RNA is a very potent method of inhibiting protein function.

 SIRNA-027 (SIRNA Therapeutics, Inc.) is a short interfering RNA that targets the VEGF receptor 1 (VEGFR-1). VEGFR-1 is found primarily on vascular endothelial cells and is stimulated by both VEGF and placental growth factor (PlGF), resulting in the growth of new blood vessels. Animal experiments have shown that both intravitreous and periocular injections of siRNA directed against VEGFR1 lead to a substantial reduction of VEGFR1 messenger RNA levels. The siRNA suppressed the development of CNV at rupture sites in Bruch's membrane and decreased retinal neovascularization in mice with oxygen-induced ischemic retinopathy. These data suggest that VEGFR1 plays an important role in the development of ocular neovascularization [62, 63].

Acuity Pharmaceuticals has also produced a siRNA called Cand5 or Bevasiranib that targets the messenger RNA of the VEGF protein itself. Animal models have shown prevention of CNV after laser-induced injury.

Bevasiranib sodium was developed for intravitreal administration and, like other intravitreal antiangiogenic agents, requires knowledge of specialized injection techniques. Following intravitreal injection, bevasiranib is well distributed within the eye and localizes to the retina [64, 65].

 Preliminary results of Phases I and II clinical trials of bevasiranib have shown promising results for the treatment of wet AMD and diabetic macular edema.

 There are various studies of different phases underway (but COBALT study is terminated). A phase III study evaluating the combination of bevasiranib and ranibizumab in wet AMD (the CARBON study) is currently underway. The purpose of this study is to compare intravitreal bevasiranib sodium as maintenance therapy for AMD following initiation of anti-VEGF therapy with three doses of ranibizumab. Preliminary clinical results indicate that the effects of bevasiranib do not appear until six weeks after the commencement of treatment, which suggests that combination therapy when using bevasiranib as an adjunct might be justified. The notion that the effect of bevasiranib is appearing late might be linked to its mechanism of action, since bevasiranib inhibits the synthesis of new VEGF, and does not eliminate existing VEGF, a direct anti-VEGF agent may be required to neutralize VEGF already present in the eye before the effects of inhibiting new VEGF synthesis are realized. Preliminary results of the carbon and cobalt studies suggested that over 30% of patients on combination ranibizumab-bevasiranib achieve at least three more lines of visual acuity than those on ranibizumab alone. The safety and efficacy of this combination awaits the full results of the ongoing clinical trials.

 Although the safety profiles and efficacy results of clinical trials are promising, the lack of available data from randomized placebo-controlled or comparative studies makes it difficult to objectively evaluate the role of bevasiranib in wet AMD therapy. It is clear from experimental and preclinical studies that anti-VEGF siRNA (either bevasiranib or similar formulations) technology is capable of downregulating VEGF production, a key goal of anti-VEGF therapy [66].

 In summary, bevasiranib exploits an interesting technology [66, 67] and may be a useful addition to the currently available drugs used to treat wet AMD. 

## 10. Anti-VEGF and Macular Edema

The DA VINCI study, a phase II study, tries to determine whether different doses and dosing regimens of intravitreal vascular endothelial growth factor (VEGF) Trap-Eye are superior to focal/grid photocoagulation in eyes with diabetic macular edema (DME). Patients were assigned to 1 of 5 treatment regimens: 0.5 mg VEGF Trap-Eye every 4 weeks; 2 mg VEGF Trap-Eye every 4 weeks, 2 mg VEGF Trap-Eye for 3 initial monthly doses and then every 8 weeks, 2 mg VEGF Trap-Eye for 3 initial monthly doses and then on an as-needed (PRN) basis, or macular laser photocoagulation. Assessments were completed at baseline and every 4 weeks thereafter. The main outcome measures were the mean change in visual acuity and central retinal thickness (CRT) at 24 weeks.

The primary results showed that intravitreal VEGF Trap-Eye produced a statistically significant and clinically relevant improvement in visual acuity when compared with macular laser photocoagulation in patients with DME.

In detail, patients in the 4 VEGF Trap-Eye groups experienced mean visual acuity benefits ranging from +8.5 to +11.4 Early Treatment of Diabetic Retinopathy study (ETDRS) letters versus only +2.5 letters in the laser group (*P* ≤ 0.0085 for each VEGF Trap-Eye group versus laser). Gains from baseline of 0+, 10+, and 15+ letters were seen in up to 93%, 64%, and 34% of VEGF Trap-Eye groups versus up to 68%, 32%, and 21% in the laser group, respectively. Mean reductions in CRT in the 4 VEGF Trap-Eye groups ranged from −127.3 to −194.5 *μ*m compared with only −67.9 *μ*m in the laser group (*P* = 0.0066 for each VEGF Trap-Eye group versus laser). VEGF Trap-Eye was generally well tolerated. Ocular adverse events in patients treated with VEGF Trap-Eye were generally consistent with those seen with other intravitreal anti-VEGF agents [68].

Also another phase II controlled clinical trial by Senger et al. [3] and Cunningham et al. [69] showed a considerably higher rate of visual gain of ≥10 letters of visual acuity for pegaptanib-treated patients compared with sham-injected patients (34% versus 10%) for patients with diabetic macular edema. The conclusion of this trial was that subjects assigned to pegaptanib had better VA outcomes, were more likely to show reduction in central retinal thickness, and were deemed less likely to need additional therapy with photocoagulation at followup.

Also smaller studies show that the intravitreal use of bevacizumab can be a safe alternative treatment and a promising option for patients suffering from DME [70, 71].

## 11. Conclusions

Numerous clinical trials are undergone to examine the comparative efficacy and safety of these anti-VEGF agents. While these studies may not detect clinical superiority of one agent over the over, larger prospective randomized trials will reassure their safety and confirm their efficacy in the future. Anti-VEGF agents are now under evaluation for other diseases such as pathologic myopia, angioid streaks, idiopathic polypoidal choroidal vasculopathy, and ocular histoplasmosis.

## Figures and Tables

**Table 1:** Ranibizumab-related adverse events followed 24-month study period.

Adverse Events at 24 months	Sham injection	Group 0.3 mg	Group 0.5 mg
Endopnthalmitis	0%	0.8%	1.3%
Uveitis	0%	1.3%	1.3%
Rhegmatogenous retinal detachment	0.4%	0%	0%
Retinal tear	0%	0.4%	0.4%
Vitreous hemorrhage	0.8%	0.4%	0.4%
Lens damage	0%	0%	0.4%

Most severe ocular inflammation			
none	87.3	83.2%	79.1%
trace	10.2	8.0%	14.6%
1+	2.5%	5.9%	3.3%
2+	0	0.8%	0.8%
3+	0	0.8%	0.8%
4+	0	1.3%	1.3%

Nonocular adverse events			

Arterial hypertension	16.1	17.2%	16.3%
Myocardial infarction	1.7%	2.5%	1.3%
Stroke	0.8%	1.3%	2.5%
Non ocular hemorrhage	5.5%	9.2%	8.8%

## References

[1] Bhisitkul RB (2006). Vascular endothelial growth factor biology: clinical implications for ocular treatments. *British Journal of Ophthalmology*.

[2] Ng EW, Adamis AP (2005). Targeting angiogenesis, the underlying disorder in neovascular age-related macular degeneration. *Canadian Journal of Ophthalmology*.

[3] Senger DR, Galli SJ, Dvorak AM, Perruzzi CA, Susan Harvey V, Dvorak HF (1983). Tumor cells secrete a vascular permeability factor that promotes accumulation of ascites fluid. *Science*.

[4] Ferrara N, Gerber HP, LeCouter J (2003). The biology of VEGF and its receptors. *Nature Medicine*.

[5] Leung DW, Cachianes G, Kuang WJ, Goeddel DV, Ferrara N (1989). Vascular endothelial growth factor is a secreted angiogenic mitogen. *Science*.

[6] Ferrara N (2004). Vascular endothelial growth factor: basic science and clinical progress. *Endocrine Reviews*.

[7] Praidou A, Androudi S, Brazitikos P, Karakiulakis G, Papakonstantinou E, Dimitrakos S (2010). Angiogenic growth factors and their inhibitors in diabetic retinopathy. *Current Diabetes Reviews*.

[8] Aiello LP, Northrup JM, Keyt BA, Takagi H, Iwamoto MA (1995). Hypoxic regulation of vascular endothelial growth factor in retinal cells. *Archives of Ophthalmology*.

[9] Karkkainen MJ, Mäkinen T, Alitalo K (2002). Lymphatic endothelium: a new frontier of metastasis research. *Nature Cell Biology*.

[10] Olofsson B, Korpelainen E, Pepper MS (1998). Vascular endothelial growth factor B (VEGF-B) binds to VEGF receptor-1 and regulates plasminogen activator activity in endothelial cells. *Proceedings of the National Academy of Sciences of the United States of America*.

[11] Bauer SM, Bauer RJ, Liu ZJ, Chen H, Goldstein L, Velazquez OC (2005). Vascular endothelial growth factor-C promotes vasculogenesis, angiogenesis, and collagen constriction in three-dimensional collagen gels. *Journal of Vascular Surgery*.

[12] Carmeliet P, Ferreira V, Breier G (1996). Abnormal blood vessel development and lethality in embryos lacking a single VEGF allele. *Nature*.

[13] Ferrara N (2002). Role of vascular endothelial growth factor in physiologic and pathologic angiogenesis: therapeutic implications. *Seminars in Oncology*.

[14] Yonekura H, Sakurai S, Liu X (1999). Placenta growth factor and vascular endothelial growth factor B and C expression in microvascular endothelial cells and pericytes. Implication in autocrine and paracrine regulation of angiogenesis. *Journal of Biological Chemistry*.

[15] Stacker SA, Caesar C, Baldwin ME (2001). VEGF-D promotes the metastatic spread of tumor cells via the lymphatics. *Nature Medicine*.

[16] Takahashi H, Shibuya M (2005). The vascular endothelial growth factor (VEGF)/VEGF receptor system and its role under physiological and pathological conditions. *Clinical Science*.

[17] Ishida S, Usui T, Yamashiro K (2003). VEGF164-mediated inflammation is required for pathological, but not physiological, ischemia-induced retinal neovascularization. *Journal of Experimental Medicine*.

[18] McColm JR, Geisen P, Hartnett ME (2004). VEGF isoforms and their expression after a single episode of hypoxia or repeated fluctuations between hyperoxia and hypoxia: relevance to clinical ROP. *Molecular Vision*.

[19] Yi X, Ogata N, Komada M (1997). Vascular endothelial growth factor expression in choroidal neovascularization in rats. *Graefe’s Archive for Clinical and Experimental Ophthalmology*.

[20] Keyt BA, Berleau LT, Nguyen HV (1996). The carboxyl-terminal domain (111–165) of vascular endothelial growth factor is critical for its mitogenic potency. *Journal of Biological Chemistry*.

[21] Ishida S, Usui T, Yamashiro K (2003). VEGF164 is proinflammatory in the diabetic retina. *Investigative Ophthalmology and Visual Science*.

[22] Storkebaum E, Carmeliet P (2004). VEGF: a critical player in neurodegeneration. *Journal of Clinical Investigation*.

[23] Jin KL, Mao XO, Greenberg DA (2000). Vascular endothelial growth factor rescues HN33 neural cells from death induced by serum withdrawal. *Journal of Molecular Neuroscience*.

[24] Sondell M, Lundborg G, Kanje M (1999). Vascular endothelial growth factor has neurotrophic activity and stimulates axonal outgrowth, enhancing cell survival and Schwann cell proliferation in the peripheral nervous system. *Journal of Neuroscience*.

[25] Papapetropoulos A, García-Cardeña G, Madri JA, Sessa WC (1997). Nitric oxide production contributes to the angiogenic properties of vascular endothelial growth factor in human endothelial cells. *Journal of Clinical Investigation*.

[26] Rakic JM, Lambert V, Devy L (2003). Placental growth factor, a member of the VEGF family, contributes to the development of choroidal neovascularization. *Investigative Ophthalmology and Visual Science*.

[27] Aiello LP, Avery RL, Arrigg PG (1994). Vascular endothelial growth factor in ocular fluid of patients with diabetic retinopathy and other retinal disorders. *New England Journal of Medicine*.

[28] Boyd SR, Zachary I, Chakravarthy U (2002). Correlation of increased vascular endothelial growth factor with neovascularization and permeability in ischemic central vein occlusion. *Archives of Ophthalmology*.

[29] Noma H, Minamoto A, Funatsu H (2006). Intravitreal levels of vascular endothelial growth factor and interleukin-6 are correlated with macular edema in branch retinal vein occlusion. *Graefe’s Archive for Clinical and Experimental Ophthalmology*.

[30] Kvanta A, Algvere PV, Berglin L, Seregard S (1996). Subfoveal fibrovascular membranes in age-related macular degeneration express vascular endothelial growth factor. *Investigative Ophthalmology and Visual Science*.

[31] Praidou A, Papakonstantinou E, Androudi S, Georgiadis N, Karakiulakis G, Dimitrakos S (2011). Vitreous and serum levels of vascular endothelial growth factor and platelet-derived growth factor and their correlation in patients with non-proliferative diabetic retinopathy and clinically significant macula oedema. *Acta Ophthalmologica*.

[32] Praidou A, Klangas I, Papakonstantinou E (2009). Vitreous and serum levels of platelet-derived growth factor and their correlation in patients with proliferative diabetic retinopathy. *Current Eye Research*.

[33] Adamis AP, Miller JW, Bernal MT (1994). Increased vascular endothelial growth factor levels in the vitreous of eyes with proliferative diabetic retinopathy. *American Journal of Ophthalmology*.

[34] Miller JW, Adamis AP, Shima DT (1994). Vascular endothelial growth factor/vascular permeability factor is temporally and spatially correlated with ocular angiogenesis in a primate model. *American Journal of Pathology*.

[35] Blaauwgeers HG, Holtkamp GM, Rutten H (1999). Polarized vascular endothelial growth factor secretion by human retinal pigment epithelium and localization of vascular endothelial growth factor receptors on the inner choriocapillaris: evidence for a trophic paracrine relation. *American Journal of Pathology*.

[36] Adamis AP, Shima DT, Tolentino MJ (1996). Inhibition of vascular endothelial growth factor prevents retinal ischemia-associated iris neovascularization in a nonhuman primate. *Archives of Ophthalmology*.

[37] Krzystolik MG, Afshari MA, Adamis AP (2002). Prevention of experimental choroidal neovascularization with intravitreal anti-vascular endothelial growth factor antibody fragment. *Archives of Ophthalmology*.

[38] Amano S, Rohan R, Kuroki M, Tolentino M, Adamis AP (1998). Requirement for vascular endothelial growth factor in wound- and inflammation-related corneal neovascularization. *Investigative Ophthalmology and Visual Science*.

[39] Ciulla T, Rosenfeld P (2009). Anti-vascular endothelial growth factor therapy for neovascular ocular diseases other than age-related macular degeneration. *Current Opinion in Ophthalmology*.

[40] Steinbrook R (2006). The price of sight-ranibizumab, bevacizumab, and the treatment of macular degeneration. *New England Journal of Medicine*.

[41] Fine SL, Berger JW, Maguire MG, Ho AC (2000). Age-related macular degeneration. *New England Journal of Medicine*.

[42] Bird AC, Bressler NM, Bressler SB (1995). An international classification and grading system for age-related maculopathy and age-related macular degeneration: the International ARM Epidemiological Study group. *Survey of Ophthalmology*.

[43] Waisbourd M, Loewenstein A, Goldstein M, Leibovitch I (2007). Targeting vascular endothelial growth factor: a promising strategy for treating age-related macular degeneration. *Drugs and Aging*.

[44] Rosenfeld PJ, Brown DM, Heier JS (2006). Ranibizumab for neovascular age-related macular degeneration. *New England Journal of Medicine*.

[45] Jager RD, Mieler WF, Miller JW (2008). Age-related macular degeneration. *New England Journal of Medicine*.

[46] De Jong PT (2006). Age-Related Macular Degeneration. *N Engl J Med.*.

[47] Gragoudas ES, Adamis AP, Cunningham ET, Feinsod M, Guyer DR (2004). Pegaptanib for neovascular age-related macular degeneration. *New England Journal of Medicine*.

[48] Vingerling JR, Dielemans I, Hofman A (1995). The prevalence of age-related maculopathy in the Rotterdam study. *Ophthalmology*.

[49] Regillo CD, Brown DM, Abraham P (2008). Randomized, double-masked, sham-controlled trial of ranibizumab for neovascular age-related macular degeneration: PIER study year 1. *American Journal of Ophthalmology*.

[50] Fung AE, Lalwani GA, Rosenfeld PJ (2007). An optical coherence tomography-guided, variable dosing regimen with intravitreal ranibizumab (Lucentis) for neovascular age-related macular degeneration. *American Journal of Ophthalmology*.

[51] Bressler NM, Chang TS, Fine JT, Dolan CM, Ward J (2009). Improved vision-related function after ranibizumab vs photodynamic therapy: a randomized clinical trial. *Archives of Ophthalmology*.

[52] Brown DM, Michels M, Kaiser PK, Heier JS, Sy JP, Ianchulev T (2009). Ranibizumab versus verteporfin photodynamic therapy for neovascular age-related macular degeneration: two-year results of the ANCHOR study. *Ophthalmology*.

[53] Holz FG, Amoaku W, Donate J (2011). Safety and efficacy of a flexible dosing regimen of ranibizumab in neovascular age-related macular degeneration: the SUSTAIN study. *Ophthalmology*.

[54] Martin DF, Maguire MG, Ying GS, Grunwald JE, Fine SL, Jaffe GJ (2011). Ranibizumab and bevacizumab for neovascular age-related macular degeneration. *New England Journal of Medicine*.

[55] Singerman LJ, Masonson H, Patel M (2008). Pegaptanib sodium for neovascular age-related macular degeneration: third-year safety results of the VEGF inhibition study in ocular neovascularisation (VISION) trial. *British Journal of Ophthalmology*.

[56] Feucht N, Matthias H, Lohmann CP, Maier M (2008). Pegaptanib sodium treatment in neovascular age-related macular degeneration: clinical experience in Germany. *Clinical Ophthalmology*.

[57] Abouammoh M, Sharma S (2011). Ranibizumab versus bevacizumab for the treatment of neovascular age-related macular degeneration. *Current Opinion in Ophthalmology*.

[58] Dixon JA, Oliver SC, Olson JL, Mandava N (2009). VEGF Trap-Eye for the treatment of neovascular age-related macular degeneration. *Expert Opinion on Investigational Drugs*.

[59] Nguyen QD, Shah SM, Hafiz G (2006). A phase I trial of an IV-administered vascular endothelial growth factor trap for treatment in patients with choroidal neovascularization due to age-related macular degeneration. *Ophthalmology*.

[60] Do DV, Nguyen QD, Shah SM (2009). An exploratory study of the safety, tolerability and bioactivity of a single intravitreal injection of vascular endothelial growth factor Trap-Eye in patients with diabetic macular oedema. *British Journal of Ophthalmology*.

[61] Barik S (2004). Development of gene-specific double-stranded RNA drugs. *Annals of Medicine*.

[62] Fire A, Xu S, Montgomery MK, Kostas SA, Driver SE, Mello CC (1998). Potent and specific genetic interference by double-stranded RNA in caenorhabditis elegans. *Nature*.

[63] Castanotto D, Rossi JJ (2009). The promises and pitfalls of RNA-interference-based therapeutics. *Nature*.

[64] Tolentino MJ, Brucker AJ, Fosnot J (2004). Intravitreal injection of vascular endothelial growth factor small interfering RNA inhibits growth and leakage in a nonhuman primate, laser-induced model of choroidal neovascularization. *Retina*.

[65] Tolentino MJ, Brucker AJ, Fosnot JY (2004). Intravitreal injection of vascular endothelial growth factor small interfering RNA inhibits growth and leakage in the nonhuman primate, laser-induced model of choroidal neovascularization. *Retina*.

[66] Garba AO, Mousa SA (2010). Bevasiranib for the treatment of wet age-related macular degeneration. *Journal of Ophthalmology and Eye Diseases*.

[67] Perkel J (2009). RNAi therapeutics: a two-year update. *Science*.

[68] Do DV, Schmidt-Erfurth U, Gonzalez VH (2011). The da VINCI study: phase 2 primary results of VEGF trap-eye in patients with diabetic macular edema. *Ophthalmology*.

[69] Cunningham ET, Adamis AP, Altaweel M (2005). A phase II randomized double-masked trial of pegaptanib, an anti-vascular endothelial growth factor aptamer, for diabetic macular edema. *Ophthalmology*.

[70] Kriechbaum K, Michels S, Prager F (2008). Intravitreal Avastin for macular oedema secondary to retinal vein occlusion: a prospective study. *British Journal of Ophthalmology*.

[71] Schaal KB, Höh AE, Scheuerle A, Schütt F, Dithmar S (2007). Bevacizumab for the treatment of macular edema secondary to retinal vein occlusion. *Ophthalmologe*.

